# Novel therapeutic approaches to simultaneously target rhinovirus infection and asthma/COPD pathogenesis

**DOI:** 10.12688/f1000research.11978.1

**Published:** 2017-10-19

**Authors:** Carmen Mirabelli, Els Scheers, Johan Neyts

**Affiliations:** 1 Laboratory of Virology and Chemotherapy, Rega Institute for Medical Research, Leuven, Belgium

**Keywords:** rhinovirus, asthma, COPD, antiviral

## Abstract

Rhinoviruses are exclusive respiratory pathogens and the etiological agents of the common cold. These viruses are increasingly reported to cause exacerbations of asthma and chronic obstructive pulmonary disease (COPD). Here, we review the role of rhinovirus infections in the pathogenesis of asthma and COPD and we discuss the current and potential future treatments. We propose that, in order to prevent exacerbations, the design of novel therapeutics should focus on directly acting antivirals but also include the design of drugs that simultaneously inhibit viral replication and alleviate symptoms of asthma and COPD.

## General introduction of rhinoviruses

Rhinoviruses (RVs) belong to the large family of *Picornaviridae* ^1^ and are grouped in three species: RV-A, RV-B, and the relatively recently characterized RV-C. Species and type assignment relies on genetic classification, in particular 13% nucleotide divergence in VP1 or more than 10% divergence in VP4/VP2 or both ^2^. This classification does not correspond to the receptor usage of RVs, the routes of entry, or the interaction with the immune system of the host ^3^. RV-A and RV-B can bind intercellular adhesion molecule 1 (ICAM-1, major group: the majority of RV-A types and all RV-B types) or low-density lipoprotein receptor (LDLR, minor group: 12 RV-A types). The receptor for RV-C15 was recently identified as the cadherin-related family member 3 (CDHR3) ^4^ and is likely shared by all members of species C, although no empirical evidence has been produced for other RV-C types. The viral cycle of RVs is exclusively cytoplasmic. The genome, a single-stranded positive RNA strain, undergoes cap-independent translation into a long polyprotein, cleaved by viral proteases 2A and 3C. Replication and encapsidation of new viral particles occur in loosely organized membrane systems bourgeoning from the Golgi apparatus ^5^ and are assisted by a number of host factors—protein kinase D, oxysterol binding protein, phosphatidylinositol 4-kinase III beta, and glutathione ^6– 8^—together with viral proteins: 3D viral polymerase and the multi-factorial 2C and 3A proteins. Lytic exit of RVs from cells may not be the sole mechanism by which viruses are released. Evidence was recently produced to support the non-lytic release of the newly produced virions within phosphatidylserine lipid-enriched vesicles ^9^.

RVs are extremely host-specific respiratory tract pathogens, mainly associated with upper respiratory tract infections in humans. RVs have long been appointed as non-harmful pathogens, and the cure for the common cold has not been considered a high-priority medical need universally. Furthermore, the development of antiviral strategies was largely hampered by the unavailability of models of infection (particularly small animal models). The extreme antigenic diversity of RVs discouraged, and still discourages, the development of vaccines. In addition, the genetic variability within the species necessitates the development of broad-spectrum, pan-RV-active antiviral drugs.

Lately, infections with RVs have been increasingly reported in the bronchial epithelium of the lower respiratory tract and even in B lymphocytes ^10^, and there are overwhelming data demonstrating that infection with these viruses causes exacerbations of asthma and chronic obstructive pulmonary disease (COPD). In the following paragraphs, we will discuss the involvement of RVs in the potential initiation or the development of these respiratory chronic syndromes (or both) and the possible therapeutic strategies to adopt for prevention and treatment.

## Asthma and rhinovirus

Asthma is a disease of the airways characterized by airflow obstruction and airway hyper-responsiveness. Depending on the severity of the disease, asthma is clinically classified as intermittent asthma or persistent asthma (mild, moderate, or severe). However, the biological definition of asthma is more complex given that more than 10 phenotypes have been described ^11^. Overall, initiation of asthma is associated with enhanced type 2 (T helper 2, Th2) or reduced type 1 immunologic responses (or both), particularly mediated by eosinophils (interleukin [IL]-5) or neutrophils (IL-8). Both eosinophils and neutrophils trigger sustained and chronic airway inflammation, which causes airway remodeling and results in decreased lung function. The majority of asthma cases have an allergic component (immunoglobulin E, IgE). Asthma attacks, clinically termed exacerbations, are mostly induced by allergen exposure and respiratory tract infections. The causes of asthma initiation are currently unknown, and both genetic and environmental factors may contribute to the establishment of the pathological condition.

### Potential role of rhinovirus in asthma initiation

Early-life human RV infection has been associated with asthma development in infants. Clinical prospective and retrospective studies demonstrated that children with RV-related wheezing illnesses present an increased risk of developing childhood asthma (Childhood Allergy Study [CAS], Childhood Origins of Asthma [COAST], Copenhagen Prospective Studies on Asthma in Childhood [COPSAC], and Tucson Children’s Respiratory Study [TCRS] studies). These findings have been extensively reviewed elsewhere ^12, 13^. The role of RV infection in the initiation of asthma remains unclear. Recently, a genome-wide association study revealed a link between a polymorphism in the CDHR3 ** gene ( *C529Y*) and early childhood asthma ^14^. Interestingly, CDHR3 is the putative receptor of the RV-C type and the CDHR3-Y _529_ polymorphism has been associated with a higher expression of the receptor at the cell surface than CDHR3-C _529_. A consequent increased susceptibility to RV-C infection could cause illness early in life, airway damage and remodeling, lung function decline, and ultimately asthma development ^15^. A convincing study by the Hershenson group recently described an increased expression of the type 2 cytokines IL-13, IL-4, and IL-5 and decreased expression of type 1 cytokine genes, interferon gamma (IFNγ) and IL12p40, immediately after RV infection in neonatal mice and not in adult mice ^16^. In addition, in these neonatal mice, RV infection induced sustained IL-13 expression, mucous metaplasia, and airway hyper-responsiveness, which are typical pathophysiologic changes described in asthmatic humans ^17^. In addition to having a potential role in asthma initiation, RV infection is known to be a major cause of asthma exacerbations.

In adults, a study of cohabiting partners, one of whom had asthma, found that although there was no difference in the frequency of RV infections, the partners with asthma exhibited greater lower respiratory symptoms and change in airway physiology ^18^. The causal link between allergic inflammation and increased susceptibility to RVs was recently proved by prophylactic treatment with an anti-IgE, omalizumab. It was shown that prophylactic treatment with omalizumab decreases fall exacerbation rates ^19^ and reduces the duration of RV infections, viral shedding, and the risk of RV illnesses ^20^ in asthmatic children and young adults. Little is known about the fundamental mechanisms responsible for the fact that asthmatics are more susceptible to RV-induced airway disease. Recent evidence points to an abnormal innate antiviral immunity (IFN dysregulation in airways), exaggerated production of inflammatory molecules such as the thymic stromal lymphopoietin, which primes Th2 responses, and an altered antibacterial host defense during acute RV infection ^21^.

## Chronic obstructive pulmonary disease and rhinovirus

COPD is a leading cause of mortality with an estimated 3 million deaths globally per year (5% of all deaths). COPD is a group of respiratory disorders characterized by a progressive, non-reversible airflow limitation, associated with a chronic inflammatory response in the lung to inhaled environmental agents, mainly cigarette smoke and biomass fuels. In contrast to asthma, COPD develops slowly and usually becomes apparent after the age of 40 years with risk for mortality from age 50 ^22^. Although it is well known that COPD is triggered by environmental factors (and mainly cigarette smoking), the underlying mechanism of individual susceptibility is not yet fully understood ^23^. The principal feature of COPD is airflow limitation by thickening of the small-airway walls and destruction of lung tissue, mainly caused by enhanced pulmonary inflammation (recruitment of macrophages, neutrophils, and CD8 ^+^ T cells), oxidative stress (augmented activity of reactive oxygen species), and a protease/antiprotease imbalance ^24– 26^. As the disease progresses, tertiary lymphoid aggregates have been shown to develop around the small airways ^24, 27^.

### Mechanisms of rhinovirus-induced exacerbation in chronic obstructive pulmonary disease

Much of the morbidity, mortality, and health-care costs of COPD is attributable to acute worsening of respiratory symptoms or exacerbations. Exacerbations are commonly caused by viral or bacterial airway infections or both. The viral pathogens responsible for 60% to 80% of all exacerbations are RV, the respiratory syncytial virus, and influenza virus ^28^. In particular, RV has been detected (by PCR) in 3.1% to 27% of COPD exacerbations ^29^. Recently, a human model to study RV-induced exacerbations of COPD formally proved a causative relationship between RV infection and COPD exacerbation ^25^. George and colleagues reported that the frequency of exacerbations correlated with RV viral loads in the sputum ^30^. The authors proposed that the mechanism of increased viral susceptibility in patients with COPD resides in the modulation of ICAM-1 on respiratory epithelial cells ^30^. Interestingly, increased expression of ICAM-1 at the surface of airway epithelial cells was also reported in response to tobacco smoke exposure ^31^. Mallia and colleagues have provided evidence that RV infection in patients with COPD is associated with recruitment of circulating T lymphocytes to the lungs and that the T-cell numbers in bronchial alveolar lavage correlate with viral load ^32^. Evidence of a direct effect of the viral protease 2A in the induction of Th1 and Th2 immune responses from CD4 T cells was also described *in vitro* ^33^. Furthermore, intranasal administration of recombinant RV protease 2A in mice resulted in an increased airway hyper-reactivity, lung inflammation, and IL-4 and IFN-γ production, suggesting a direct role of 2A in the Th1 and Th2 hyper-response during COPD exacerbations ** ^33^.

## Antiviral strategies for rhinovirus infection

**Figure 1.**
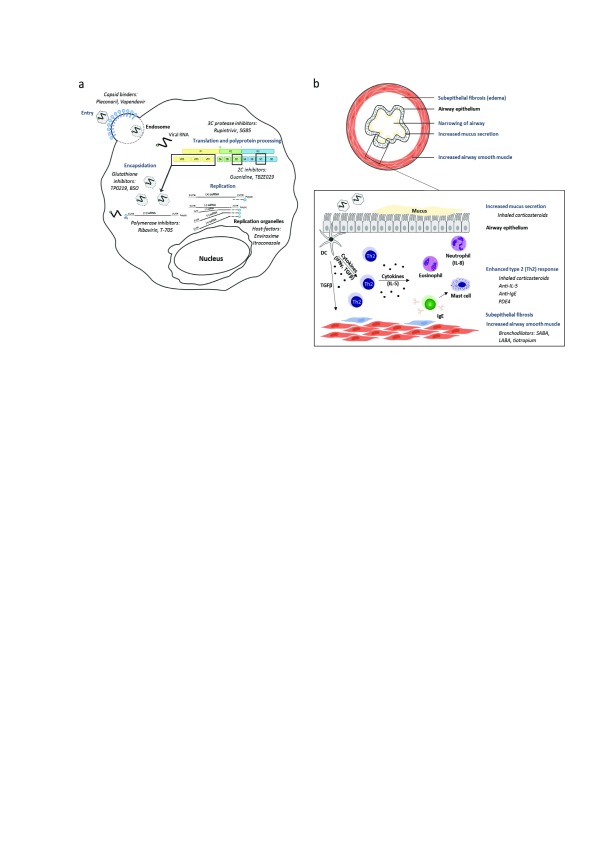
Rhinovirus inhibitors and asthma/COPD therapeutics. ( **A**) Schematic representation of the rhinovirus replication cycle, including different classes of inhibitors and representative examples thereof (italics). ( **B**) Cross-section of a bronchial tube with asthma/COPD pathogenesis and detail of the epithelial barrier illustrating the enhanced type 2 (Th2) immunologic response leading to airway remodeling. Relevant selections of therapeutics used for asthma/COPD treatment are indicated in italics. DC, dendritic cell; IFNγ, interferon gamma; IL, interleukin; LABA, long-acting B2-agonist bronchodilator; PDE4, phosphodiesterase 4; SABA, short-acting B2-agonist bronchodilator; ssRNA, single-stranded RNA; TGFβ, transforming growth factor-beta; UTR, untranslated region.

The genetic diversity of RVs has so far hampered the development of both prophylactic and therapeutic antiviral strategies. More than 150 serotypes of RV are known, and cross-protection appears to be limited. The slow and disappointing efforts to develop RV vaccines have been exhaustively reviewed elsewhere ^34, 35^. For the development of small antiviral molecules, each step of RV viral cycle could theoretically represent a target, and several candidates have been described over the years. Comprehensive reviews on efforts to develop small-molecule inhibitors of RV/enterovirus replication have been published ^36, 37^. The most-studied compounds are presented in Figure 1A.

Capsid binders are inhibitors of viral entry, and the most/best-studied series is composed of the so-called WIN compounds. These molecules bind a hydrophobic pocket located under the floor of the canyon, which is the depression on the viral capsid surface involved in cell receptor binding. The drug fits the pocket and stabilizes the virion by pushing the floor of the canyon upwards, thereby preventing capsid conformational changes necessary for virus uncoating ^38^. The potential efficacy of three WIN-capsid binders (that is, pirodavir, pleconaril, and vapendavir) against RV infections in humans was assessed by the Janssen Research Foundation, Schering-Plough (nasal formulation by ViroPharma), and Biota, respectively ( 39, clinicaltrial.gov). Pirodavir was the first small-molecule shown to prevent experimental RV infection in a human challenge model. However, the compound was active only when administered six times daily and when treatment was initiated within 10 minutes after RV challenge. When start of treatment was delayed until 24 or 48 hours after challenge, the antiviral effect was no longer observed ^40^. Pleconaril failed the first clinical trial with an oral formulation that was given three times per day when treatment started 24 hours after challenge (only 1 to 1.5 days of reduction in symptom alleviation time was demonstrated) ^41^. Furthermore, drug-resistant RV strains were identified in 24% of enrolled patients: 13% of patients were naturally resistant at baseline, and 11% exhibited a reduced susceptibility by day 5 of treatment ^39, 42^. A phase II study, which used an intranasal formulation of pleconaril, failed to show a statistically significant result for RV-positive participants either with or without asthma exacerbations ^39^. Recently, vapendavir in a phase IIb clinical trial (SPIRITUS) resulted in a statistically significant antiviral effect (RV PCR-negative) for patients who received the compound within 24 hours after onset of symptoms. However, no reduction in secondary endpoints (lung function and reduction in asthma exacerbations) was noted (Biota website). Overall, although capsid binders are attractive and potent early-stage inhibitors of RV replication *in vitro*, problems in pharmacodynamics, *in vivo* efficacy, and resistance development were reported. In addition, with the recent resolution of the capsid structure, it was concomitantly demonstrated that RV-C species lack the hydrophobic pocket, the binding target of capsid binders. Therefore, pan-RV coverage cannot be achieved with this specific class of compounds ^43^. In contrast to capsid binders, the pan-enterovirus 3C protease inhibitor rupintrivir, a Michaelis acceptor, exerts low nanomolar potency against a large panel of RV-A and RV-B serotypes *in vitro*. Moreover, activity was demonstrated against RV-C in a replicon system ^44^. Other protease inhibitors have been successfully developed as antiviral drugs for the treatment of infections with the human immunodeficiency virus and more recently the hepatitis C virus (HCV). However, the antiviral effect of the orally available analogue of rupintrivir, named “Compound 1”, was investigated in naturally infected RV patients and no reduction in disease severity or viral load was noted. Hence, further development was halted ^39^, and the effect of the treatment was not evaluated in the context of patients with asthma or COPD.

A safe and efficient antiviral for the treatment of RVs would be appreciated by many, including otherwise-healthy patients with common cold, to reduce the time of symptoms and, in turn, reduce the costs of school/work productivity loss. But mostly, a highly efficient therapy is needed for the treatment or prophylaxis of RV infections in those with underlying conditions such as asthma and COPD. Unfortunately, so far, there has been no proof of concept that a direct-targeting anti-RV treatment could prevent exacerbations and alleviate asthma or COPD symptoms. In the next paragraphs, we will discuss the potential design and development of combination therapies, which may combat RV infection/replication and asthma/COPD symptoms simultaneously and constitute an interesting strategy in patients with compromising chronic conditions.

## Current treatment for asthma and chronic obstructive pulmonary disease

Asthma is a progressive pathologic condition, which generally manifests before 12 years of age. For intermittent episodes of asthma, patients undergo short-acting B2-agonist bronchodilator (SABA) treatment. For persistent asthma, inhaled corticosteroids (ICSs), the most effective anti-inflammatory medication, are coupled with SABAs or long-acting B2-agonist bronchodilators (LABAs), according to the severity of the disease. In case of uncontrolled severe asthma, tiotropium (patients > 12 years old, exacerbated), anti-IgE (patients > 6 years old), or anti-IL-5 (patients > 12 years old, eosinophilic asthma) is added ^45^. Patients with COPD differ markedly from patients with asthma: the former are older and mostly current or ex-smokers and have impaired lung function and their airflow obstruction is not reversible. Current treatments for COPD exacerbations consist of supportive therapies: SABA or LABA in addition to ICS according to the severity of disease. In the case of severe COPD, oral phosphodiesterase 4 inhibitors and azithromicyn are prescribed ^46^. Tiotropium is also used for maintenance therapy during stable COPD to reduce symptoms and the frequency and severity of exacerbations ^46, 47^. No new therapeutics have been developed over the past three decades, and the use of antibiotics for COPD is debated given the emergence and spread of antibiotic resistance. The main therapeutics for asthma and COPD are illustrated in Figure 1B.

## Rhinovirus infection interferes with the current treatment for asthma and chronic obstructive pulmonary disease

Glucocorticoids (GCs), belonging to the ICS family of drugs, are a highly effective group of anti-inflammatory agents, which are widely used in the treatment of chronic inflammatory airway diseases, including asthma and COPD. However, results from clinical trials suggest that respiratory viral infections affect GC therapy ^48– 50^. In particular, RV-induced exacerbations of asthma and COPD have been shown to be refractory to the anti-inflammatory effect of GCs ^50^. Xia and colleagues suggested the mechanism of reduced sensitivity through an airway epithelium model. RV infection appeared to increase the expression and activity of transforming growth factor-beta, which in turn induced GC insensitivity ^51^.

Interference of viral infection on current treatment was also reported in the context of budesonide or formoterol treatment (or both), two common LABAs. *In vitro* data on RV-stimulated peripheral blood mononuclear cells suggest that the combination of budesonide and formoterol inhibits RV-induced upregulation of CXCL10 and in turn has a negative effect on antiviral responses ^52^. Indeed, IFN-α secretion and expression of the type I IFN-inducible genes were enhanced, suggesting a negative interference of drugs on RV infection (less protection and more infection). In contrast, when bronchial epithelial cells were infected with RV, budesonide/formoterol treatment did not induce a significant inhibitory effect on CXCL10 secretion or type I and III IFN gene induction, suggesting that the drugs could have a beneficial effect on RV-induced asthma exacerbations (more protection and less infection) ^53^. It remains to be elucidated which of the findings is relevant to the situation in patients.

Azithromycin is a macrolide antibiotic used for the treatment of COPD since it shows a reduction in exacerbation frequency together with an improvement in quality of life of patients with COPD ^54^. When primary bronchial epithelial cells from COPD donors or healthy individuals were treated with azithromycin 24 hours before infection with RV, azithromycin transiently induced increases of IFNβ, IFNλ, and RIG-I expression. After infection, azithromycin augmented RV-induced expression of IFN- and RIG-I like-helicases in cells derived from patients with COPD and this decreased viral load. This effect was not observed in cultures obtained from healthy individuals. These data support azithromycin’s emerging role in the prevention of exacerbations of COPD ^55^.

## Conclusion and future perspective

The aforementioned examples point to the importance of studying the potential interference between RV infection and asthma/COPD treatment agents. New clinical trials take into consideration this problem of interference (clinicaltrial.gov). Therefore, the efficacy of (a) OC459, an antagonist of the prostaglandin D2 receptor 2 (known to stimulate the *in vitro* chemotaxis of Th2 cells and leukocytes and to release the granule content of eosinophils and basophils), and (b) CNTO 3157, an antagonist of Toll-like receptor 3 (which mediates antiviral response and is involved in asthma exacerbation and development), is now being explored in the context of RV infection in humans. When human RV challenge experiments are not possible or not ethical (in the case of patients with severe asthma/COPD symptoms), physiologically relevant *in vitro* models should be used. In particular, airway epithelium culture derived from healthy patients or patients with underlying chronic respiratory syndromes should be collected and differentiated *in vitro*. Moreover, the strain of RV to infect such cultures should be clinically relevant. Although they are highly prevalent in RV-induced exacerbation of asthma and COPD, little is known about the effect of RV-C species. Mechanistic studies addressing the potential differences of these strains in the context of asthma/COPD are awaited and the results should be directly compared with those obtained with the better-studied RV-A strains.

In the development of novel therapeutic approaches, efforts should be addressed toward dual-target molecules, which inhibit RV replication and target the underlying pathogenesis of asthma and COPD at the same time. Dual-target therapies are often considered farfetched and unrealistic. However, the interesting mechanism of action of azithromycin demonstrates that dual-target therapies can be achieved ^55^. IFN signaling could be an important target to alleviate asthma symptoms but also to prime the cells and subsequently prevent RV infection. Besides targeting IFN-associated pathways, other cellular targets may be used to develop synergistically acting therapeutics. We recently reported on a class of broad-spectrum enterovirus/RV inhibitors that are also correctors of the cystic fibrosis transmembrane conductance regulator (CFTR) folding ^56^. Deficiencies in CFTR are associated with more than 90% of cystic fibrosis cases. Similarly, cytokines such as the thymic stromal lymphopoietin or its receptor are pivotal in the pathophysiology of asthma (Th2-upstream activator) and are becoming an attractive cellular target for the treatment of asthma ^57^. Multi-target drug design could be employed to identify chemical scaffolds with dual activity as antiviral and anti-asthmatic compound.

The development of directly acting antivirals with an additional effect on exacerbations of asthma and COPD should also be explored. To that end, the RV 2A protease, which is an essential protein for viral replication, may be a potentially interesting target. Indeed, as previously mentioned, the expression of RV 2A was reported to induce Th1- and Th2-mediated inflammation *in vitro* and *in vivo*, suggesting a direct role of 2A in COPD exacerbations ** ^33^. Highly potent and directly acting antivirals have been developed in recent years for HCV, which, similarly to RV, is a positive single-stranded RNA virus. Today, treatment of chronic HCV is extremely successful and most patients are cured from the infection and are no longer at risk of death from chronic liver cirrhosis and hepatocellular carcinoma. Likewise, highly potent inhibitors for RV, to be used either prophylactically or therapeutically ^58^, may be sufficient to cure the infection and block the progression of underlying chronic conditions such as asthma and COPD. We propose that, together with direct pan-RV antiviral strategies, novel multi-target or combination approaches that simultaneously interfere with viral replication and the pathogenesis (of exacerbations) of asthma and COPD should be rationally designed and developed.

## Abbreviations

CDHR3, cadherin-related family member 3; CFTR, cystic fibrosis transmembrane conductance regulator; COPD, chronic obstructive pulmonary disease; GC, glucocorticoid; HCV, hepatitis C virus; IgE, immunoglobulin E; ICAM-1, intercellular adhesion molecule 1; ICS, inhaled corticosteroid; IFN, interferon; IL, interleukin; LABA, long-acting B2-agonist bronchodilator; RV, rhinovirus; SABA, short-acting B2-agonist bronchodilator; Th2, T helper 2.
